# An Optimization Method Tracking EMG, Ground Reactions Forces, and Marker Trajectories for Musculo-Tendon Forces Estimation in Equinus Gait

**DOI:** 10.3389/fnbot.2019.00048

**Published:** 2019-07-16

**Authors:** Florent Moissenet, Colombe Bélaise, Elodie Piche, Benjamin Michaud, Mickaël Begon

**Affiliations:** ^1^Centre National de Rééducation Fonctionnelle et de Réadaptation–Rehazenter, Luxembourg, Luxembourg; ^2^Laboratory of Simulation and Movement Modeling, School of Kinesiology and Exercise Sciences, Université de Montréal, Montreal, QC, Canada; ^3^Sainte-Justine Hospital Research Center, Montreal, QC, Canada

**Keywords:** musculoskeletal modeling, direct multiple shooting, co-contraction, musculo-tendon forces, neuromusculoskeletal simulations

## Abstract

In the context of neuro-orthopedic pathologies affecting walking and thus patients' quality of life, understanding the mechanisms of gait deviations and identifying the causal motor impairments is of primary importance. Beside other approaches, neuromusculoskeletal simulations may be used to provide insight into this matter. To the best of our knowledge, no computational framework exists in the literature that allows for predictive simulations featuring muscle co-contractions, and the introduction of various types of perturbations during both healthy and pathological gait types. The aim of this preliminary study was to adapt a recently proposed EMG-marker tracking optimization process to a lower limb musculoskeletal model during equinus gait, a multiphase problem with contact forces. The resulting optimization method tracking EMG, ground reactions forces, and marker trajectories allowed an accurate reproduction of joint kinematics (average error of 5.4 ± 3.3 mm for pelvis translations, and 1.9 ± 1.3° for pelvis rotation and joint angles) and ensured good temporal agreement in muscle activity (the concordance between estimated and measured excitations was 76.8 ± 5.3 %) in a relatively fast process (3.88 ± 1.04 h). We have also highlighted that the tracking of ground reaction forces was possible and accurate (average error of 17.3 ± 5.5 N), even without the use of a complex foot-ground contact model.

## Introduction

Walking is often considered to be the most important activity in daily living (Chiou et al., 1985). The ability to move without pain, fatigue, or major gait deviation is closely related to quality of life (Cuomo et al., 2007; van Schie, 2008). Many neuro-orthopedic pathologies (e.g., cerebral palsy, stroke) induce impairments (i.e., paresis, muscle overactivity, soft tissue contractures, and bone deformities) that compromise normal movement. Consequently, the goal of many therapeutic interventions is to minimize gait deviations in patients. In order to improve these interventions, understanding the possible mechanisms of these gait deviations, and being able to identify the causal motor impairments is of primary importance (Davids et al., 2004; Gough and Shortland, 2008; Wren et al., 2011). Currently, the relationship between motor impairments and gait deviations is unclear (Bonnefoy-Mazure et al., 2016), and there is a lack scientific evidence for these relationships due to the inherent complexity of the human neuromusculoskeletal system during dynamic tasks such as walking (Armand et al., 2017). Compared to existing approaches (e.g., pathologic models, experimental procedures with human subjects, robots with human-like gait), *in silico* neuromusculoskeletal simulations of normal and pathological gait could provide additional insight into gait deviations (Armand et al., 2017). The advantage of neuromuscular simulations as a method is that large numbers of simulations may be performed relatively quickly, and without the ethical issues involved with performing invasive and lengthy experiments in vulnerable patient groups such as those with neuromuscular deficits.

To date, simulations reported in the literature are often limited to the analysis of the consequences of isolated impairments on gait such as muscle weakness (van der Krogt et al., 2009; Thompson et al., 2013) or muscle spasticity (Jansen et al., 2014). Most of these studies only report possible muscular compensations (adaptations) that occur due to muscular redundancy–results are achieved by tracking the normal gait kinematics and then applying a perturbation to the model. Very few studies have been based on a numerical framework allowing kinematic adaptations in response to more varied perturbations. Within the TLEMsafe project (https://www.tlemsafe.eu), Fluit et al. (2014b) combined an optimized inverse model and a ground reaction force predictive model to simulate lower limb kinematics after the removal of the rectus femoris and the vastus lateralis from the model. Santos et al. (2017) also proposed a numerical framework based on direct collocation and an optimal control package to simulate lower limb kinematics after the introduction of a weakening of the triceps surae and the tibialis anterior, or after increasing the ankle joint stiffness. These two approaches represent a first step toward the simulation of pathological gait. However, neither were able to reproduce muscle co-contractions. While this capacity was not necessarily needed in these studies, this feature is essential to establish a pathological gait simulator that would be able to reproduce physiological gait adaptations biofidelically.

From a methodological point of view, inverse dynamics-based approaches (such as static optimization) are commonly used due to their computational efficiency (Erdemir et al., 2007), but are not appropriate for predictive simulations. Moreover, static optimization method underestimates or neglects antagonist co-contractions unless hybrid approaches are used (Brookham et al., 2011; Son et al., 2012). On the other hand, forward dynamics-based approaches are often criticized for being time-consuming–several studies report convergence times in the hundreds of hours range (Anderson and Pandy, 2001). Despite this disadvantage, these methods have the potential to predict new movements, such as an adaptation in response to a perturbation. For example, state-of-the-art algorithms used in conjunction with existing musculoskeletal models–like direct collocation (Santos et al., 2017) and direct multiple shooting (Bélaise et al., 2018a,b)—can be used to solve forward dynamics problems in a timely manner. Recently, Bélaise et al. (2018a,b) introduced an EMG-marker tracking optimization method to predict musculo-tendon forces in a co-contraction case. Based on simulated datasets of upper limb movements, the authors showed the importance of tracking both marker trajectories and EMG, in particular to reproduce muscle co-contractions. To the best of our knowledge, such an approach has never been applied on experimental gait records with muscle co-contractions.

The objective of our project is to establish a computational framework appropriate for predictive simulations of healthy and pathological gait, that is able to reproduce muscle co-contractions, and that allows for the introduction of various kind of perturbations on the model (e.g., therapy-related, surgery-related, pathology-related perturbations). This preliminary study represents a first step toward this project by adapting the computational framework proposed by Bélaise et al. (2018a,b) to a lower limb musculoskeletal model during gait. This framework has been tested for this purpose on a type of pathological gait known as equinus gait.

## Methods

### Lower Limb Musculoskeletal Model

**Figure 1:**
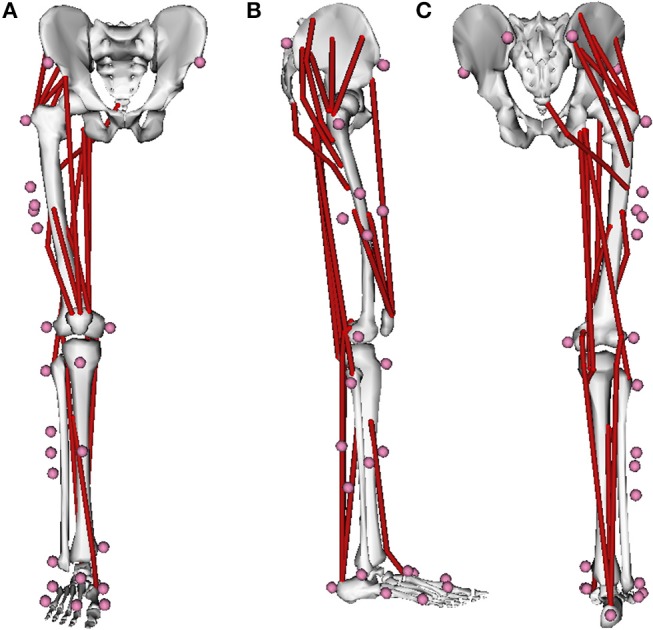
Anterior **(A)**, lateral **(B)**, and posterior **(C)** views of the right lower-limb musculoskeletal model derived from OpenSim [Lower Extremity Model, OpenSim (Delp et al., 1990)] and adapted to the *bioRBD* musculoskeletal modeling package (https://github.com/pyomeca/biorbd). Red lines and pink dots represents the 17 Hill-type muscle lines of action and the 26 markers related to this model, respectively.

A generic three-dimensional musculoskeletal model of the lower limb [Lower Extremity Model, OpenSim (Delp et al., 1990)] was adapted for our study (Figure 1). This model consists of five rigid segments: the pelvis, right thigh, patella, shank, and foot. Twenty-six markers were associated with these segments by virtual palpation to reproduce the experimental marker locations (Table 1; see section Dataset). To simplify the dynamic optimizations to a two-dimensional motion in this preliminary study, the original degrees of freedom (DoF) were reduced to three DoFs for the pelvis-ground joint (vertical translation, translation in the direction of walking, pelvis tilt) and one DoF (flexion-extension modeled as a hinge joint) at the hip, knee, and ankle joints. Joints were actuated by the muscle torques resulting from 17 muscle lines of action (Table 2), and the pelvis DoFs were actuated by three generalized forces applied on the pelvis. The path, optimal length, maximal isometric force, tendon slack length, and pennation angle of each muscle lines of action were derived from the original model (Delp et al., 1990).

**Table 1:** List of the 26 markers used in this study.

**Abbreviations**	**Palpation details**	**Related segments**
L_IAS	Left anterior-superior iliac spine	Pelvis
L_IPS	Left posterior-superior iliac spine	Pelvis
R_IPS	Right posterior-superior iliac spine	Pelvis
R_IAS	Right anterior-superior iliac spine	Pelvis
R_FTC	Right greater trochanter	Thigh
R_Thigh_Top	Superior marker of the thigh cluster	Thigh
R_Thigh_Down	Inferior marker of the thigh cluster	Thigh
R_Thigh_Front	Anterior marker of the thigh cluster	Thigh
R_Thigh_Back	Posterior marker of the thigh cluster	Thigh
R_FLE	Right lateral femoral epicondyle	Thigh
R_FME	Right medial femoral epicondyle	Thigh
R_FAX	Right fibula head	Shank
R_TTC	Right tibial tuberosity	Shank
R_Shank_Top	Superior marker of the shank cluster	Shank
R_Shank_Down	Inferior marker of the shank cluster	Shank
R_Shank_Front	Anterior marker of the shank cluster	Shank
R_Shank_Tibia	Additional marker of the shank cluster on the tibia	Shank
R_FAL	Right lateral tibial malleolus	Shank
R_TAM	Right medial tibial malleolus	Shank
R_FCC	Right posterior calcaneus	Foot
R_FM1	Right 1st distal metatarsal head	Foot
R_FMP1	Right 1st proximal metatarsal head	Foot
R_FM2	Right 2nd distal metatarsal head	Foot
R_FMP2	Right 2nd proximal metatarsal head	Foot
R_FM5	Right 5th distal metatarsal head coordinates	Foot
R_FMP5	Right 5th proximal metatarsal head coordinates	Foot

*Palpation details used to place experimental reflective cutaneous markers (as well as virtual markers by virtual palpation) and the related segment are also mentioned*.

**Table 2:** List of the 17 Hill-type muscle lines of action included in the model.

**Abbreviations**	**Muscle lines of action**	**Details**	**Joints crossed**	**Available EMG**
R_GLUT_MAX1	Gluteus maximus (1)	Anterior fibers	Hip	X
R_GLUT_MAX2	Gluteus maximus (2)	Lateral fibers	Hip	
R_GLUT_MAX3	Gluteus maximus (3)	Posterior fibers	Hip	
R_GLUT_MED1	Gluteus medius (1)	Anterior fibers	Hip	
R_GLUT_MED2	Gluteus medius (2)	Lateral fibers	Hip	X
R_GLUT_MED3	Gluteus medius (3)	Posterior fibers	Hip	
R_SEMIMEM	Semimembranosus	/	Hip, knee	
R_SEMITEN	Semitendinosus	/	Hip, knee	X
R_BI_FEM_LH	Biceps femoris	Long head	Hip, knee	X
R_RECTUS_FEM	Rectus femoris	/	Hip, knee	X
R_VAS_MED	Vastus medialis	/	Knee	X
R_VAS_INT	Vastus intermedius	/	Knee	
R_VAS_LAT	Vastus lateralis	/	Knee	
R_GAS_MED	Gastrocnemius medialis	/	Knee, ankle	X
R_GAS_LAT	Gastrocnemius lateralis	/	Knee, ankle	
R_SOLEUS	Soleus	/	Ankle	X
R_TIB_ANT	Tibialis anterior	/	Ankle	X

*The muscles abbreviations, the joint(s) they cross and the related electromyographic (EMG) signals (when available) are also mentioned*.

Segment lengths were scaled to the dataset used in this study (see section Dataset) using OpenSim 3.3 (Delp et al., 2007) by minimizing the distance between experimental and model markers placed on bony landmarks (Table 1). All components of the model that depend on bone lengths (e.g., muscle attachment points, optimal fiber length), segment masses, and inertial parameters were also scaled. The resulting scaled model was transferred to the *bioRBD* musculoskeletal modeling package (https://github.com/pyomeca/biorbd) based on the Rigid Body Dynamic Library (Felis, 2017). This model was defined by 29 states (six generalized joint positions and their six related velocities, 17 muscle activations) and 20 controls corresponding to the 17 muscle neural excitations plus three generalized forces driving the three pelvis DoFs.

### Equations of Motion and Activation Dynamics

The generalized accelerations $\ddot{\text{q}}$ of the rigid multibody system were computed using a forward dynamics approach for given generalized joint positions **q**, joint velocities $\overset{∙}{\text{q}}$, and generalized forces **τ**:

$$\begin{array}{l} \overset{..}{q}=M{\left(q\right)}^{-1}\left(\text{τ}\left(q,\;\dot{q},\;a,\;e\right)+C{\left(q\right)}^{T}R-N\left(q,\;\dot{q}\right)\dot{q}\;-\;G\left(q\right)\right)\\ \;s.t.\;C\left(q\right)\overset{..}{q}\;+\dot{\;C}\left(q\right)\dot{q}\;=\;0\;and\;C\left(q\right)\dot{q}\;=\;0 \end{array}$$

where **M** is the inertia matrix, **C** is the external contact Jacobian matrix, **R** is the Lagrange multipliers vector corresponding to the ground reaction forces (GRF), **N** is the non-linear effects (Coriolis and centrifugal forces) vector, and **G** is the gravity effects vector. It was assumed that contact points have a null acceleration and velocity throughout the entire contact phase. In line with equinus gait, one fixed contact point was defined on the forefoot for the entire contact phase (see section Dynamic Optimizations). Generalized forces were divided into $\tau_{\text{1}}={\left[\tau_{\text{11}}{\;\tau }_{\text{12}}{\;\tau }_{\text{13}}\right]}^{T}$ driving the three pelvis DoFs, and $\tau_{\text{2}}=\frac{\partial {\text{L}}_{\text{mt}}}{\partial \text{q}}{\text{F}}_{\text{mt}}$ corresponding to the net joint torques due to the musculo-tendon forces **F**_**mt**_, where $\frac{\partial {\text{L}}_{\text{mt}}}{\partial \text{q}}$ is the generalized muscular lever arms matrix and **L**_**mt**_ the vector of muscle line of action lengths. **F**_**mt**_ were computed from muscle activations **a** using a Hill-type muscle model with a generic force-length-velocity relation *f* (Zajac, 1989):

$$F_{mt}\left(q,\;\dot{q},\;a\right)\;=\;af\left(F_{mt}^{0},\;l_{m},\;v_{m}\right),$$

where ${\text{F}}_{\text{mt}}^{\text{0}}$ is the maximal isometric forces vector, **l**_**m**_ is the muscle fiber lengths vector, and **v**_**m**_ is the muscle fiber velocities vector. Again, musculo-tendon forces were divided into **F**_**mt1**_ (with related activations **a**_**1**_ and excitations **e**_**1**_), corresponding to the muscles for which electromyographic (EMG) records were available, and **F**_**mt2**_ (with related activations **a**_**2**_ and excitations **e**_**2**_) where EMG measurements were unavailable. Muscle activation dynamics was implemented as a set of first-order differential equations (Buchanan et al., 2004):

$$\dot{a}\left(t,\;e\left(t\right),\;a(t)\right)=\{\begin{array}{c} \frac{\left(e(t)-\;a\left(t\right)\right)}{t_{act}\left(0.5+1.5\;a(t)\right)},\;e\left(t\right)>\;a(t)\\ \;\frac{e(t)-\;a(t)}{t_{deact}}\left(0.5+1.5\;a(t)\right),\;e\left(t\right)\leq \;a(t) \end{array}$$

where **e**(*t*) is the muscle neural excitations at time *t*. Time constants t_*act*_ and t_*deact*_ (for activation and deactivation) were set at 10 and 40 ms, respectively (Thelen et al., 2003).

### Dynamic Optimizations

As proposed in Bélaise et al. (2018a), controls and state variables were simultaneously optimized using an EMG-marker tracking optimization process. Because the model has a reduced muscular redundancy and because a generic model was used as opposed to a subject-specific model, the optimal maximal isometric forces were also identified during this process.

The optimization consisted of the minimization of the differences between predicted **M**_**p**_ and measured **M**_**m**_ marker trajectories in the sagittal plane, and between predicted **e**_**1p**_ and measured **e**_**1m**_ (EMG envelop) muscle neural excitations (corresponding to the muscles for which EMG records are available). This tracking optimization was extended to the minimization of the differences between predicted **R**_**p**_ and measured **R**_**m**_ GRF in the sagittal plane. This tracking was necessary to impose physiologic generalized forces to the pelvis (**τ**_**1**_), i.e., generalized forces that compensate for the missing upper part of the body and contralateral lower limb.

To predict the activity of the muscles for which tracking was not possible (i.e., muscles for which EMG records were not available), the objective function *J* was written to find the least squared muscle activations **a**_**2**_ that produced the prescribed marker trajectories, muscle neural excitations, and GRF (during stance phase only):

$$\begin{array}{l} J=\overset{N_{i}}{\underset{1}{\sum }}\left(w_{\text{M}}||{\text{M}}_{p}-{\text{M}}_{m}|{|}^{2}+w_{e}||{\text{e}}_{1p}-{\text{e}}_{1m}|{|}^{2}+w_{\text{R}}||{\text{R}}_{p}-{\text{R}}_{m}|{|}^{2}\right)\\ \;+w_{L}\int_{0}^{T_{i}}{{\text{a}}_{2}\left(t\right)}^{2}dt\\ where\;w_{\text{R}}||{\text{R}}_{p}-{\text{R}}_{m}|{|}^{2}=0\;when\;\text{i}=2,\;i.e.,\;the\;swing\;phase. \end{array}$$

where *w*_M_, *w*_e_, *w*_R_, and *w*_L_ are weighting factors adjusted to the relative importance of each term, *T*_*i*_ is the duration of the current stage (see section Simulations) and *N*_*i*_ is the related number of time frames.

This objective function was minimized under three sets of constraints. First, boundary conditions were applied on the state and the control variables. In this study, the range of motion of each DoF and related velocities were set to physiologic values (Table 3), while activations and excitations were bounded between 0 and 1. Second, the velocity of the contact point was constrained to be null at the first frame and its acceleration to be null at each time frame (see section Equations of Motion and Activation Dynamics). Third, periodicity was ensured by constraining the first and last time point of the cycle to have similar values in terms of hip, knee, ankle joint angles, and velocities, pelvis velocities, muscle excitations, and GRF.

**Table 3:** Boundaries constraints applied during the optimization process to each degree of freedom and related velocities.

**Abbreviations**	**Variables**	**Min**.	**Max**.
PELVIS_TRANS_X	Pelvis ant. (+)/post. (–) translation (m)	−10.00	10.00
PELVIS_TRANS_Y	Pelvis sup. (+)/inf. (–) translation (m)	−0.50	1.50
PELVIS_ROT_Z	Pelvis ant. (–)/post. (+) tilt (°)	−45.00	45.00
R_HIP_ROT_Z	Hip flex. (+)/ext. (–) (°)	−20.00	60.00
R_KNEE_ROT_Z	Knee flex. (–)/ext. (+) (°)	−90.00	5.00
R_ANKLE_ROT_Z	Ankle dorsi. (+)/plantarflex. (–) (°)	−50.00	20.00
PELVIS_TRANS_VX	Pelvis ant. (+)/post. (–) linear velocity (m.s^−1^)	0.50	1.50
PELVIS_TRANS_VY	Pelvis sup. (+)/inf. (–) linear velocity (m.s^−1^)	−0.50	0.50
PELVIS_ROT_VZ	Pelvis ant. (–)/post. (+) tilt angular velocity (°.s^−1^)	−100	100
R_HIP_ROT_VZ	Hip flex. (+)/ext. (–) angular velocity (°.s^−1^)	−300	300
R_KNEE_ROT_VZ	Knee flex. (–)/ext. (+) angular velocity (°.s^−1^)	−300	300
R_ANKLE_ROT_VZ	Ankle dorsi. (+)/plantarflex. (–) angular vel. (°.s^−1^)	−300	300

### Simulations

Each dynamic optimization was solved using a direct multiple shooting algorithm with MUSCOD-II (Leineweber et al., 2003). Three phases were defined in the gait step: (1) the stance phase (with an external contact between foot and ground), (2) the swing phase (no external contact), and (3) the first frame of the next stance phase following the impact between the foot and the ground. These stages were divided into 25, 25, and 1 multiple shooting intervals, respectively. For the sake of simplicity, the first stage started just after the collision impact between the foot and the ground. The duration of each stage was fixed to the measured value.

The initial guess was set to the measured values for the joint positions and velocities, 1% for all activations and excitations, and 0 for the controls corresponding to the generalized forces related to the pelvis DoFs. Weighting factors were set to *w*_M_ = 30 (except for the foot markers for which *w*_M_ = 50 to ensure the correct position of the contact point), *w*_e_ = 1, *w*_R_ = 0.05, and *w*_L_ = 1. These weighting factors were adjusted empirically to set values around 1, in order to ensure optimization convergence and produce simulation results close to the experimentally measured data.

### Dataset

The previously defined method was evaluated on a dataset of emulated equinus gait. All data were recorded on a healthy volunteer (male, 35 years old, 165 cm, 66 kg) without any neuro-orthopedic conditions. This participant gave written informed consent prior to his inclusion and the protocol was conformed to the Declaration of Helsinki and approved by the National Research Ethics Committee of Luxembourg (201805/01).

The 3D trajectories of 26 reflective cutaneous markers (bilateral iliac anterior and posterior spines, right leg great trochanter, medial and lateral femoral epicondyles, peroneal head, tibial tuberosity, medial and lateral malleoli, 1st, 2nd, and 5th proximal and distal metatarsal heads, calcaneum, completed by a four-marker cluster on the thigh and on the shank) (Figure 1) were recorded using a 10-camera optoelectronic system (OQUS-4, Qualisys AB, Sweden) sampled at 200 Hz. Markers were placed by anatomical palpation (Table 1) following the recommendation of van Sint Jan (2007) by an experienced user. GRF and moments were recorded using two side-by-side force plates (OR6-5, AMTI, USA) sampled at 2,000 Hz. The EMG activity of nine right leg muscles (tibialis anterior, soleus, gastrocnemius medialis, vastus medialis, rectus femoris, semitendinosus, biceps femoris long head, gluteus medius, gluteus maximus) was collected with a wireless electromyographic system (DTS clinic, Noraxon, USA) sampled at 2,000 Hz. The EMG surface electrodes were placed following the recommended standard of the Surface EMG for a Non-Invasive Assessment of Muscles (SENIAM) project (Hermens et al., 2000).

All data were imported under Matlab (R2018a, The MathWorks, USA) using the *ezc3d* package (https://github.com/pyomeca/ezc3d). Marker trajectories were interpolated when necessary using a cubic spline and smoothed by a 4th order low-pass Butterworth filter with a cutoff frequency of 6 Hz. Generalized kinematics ($q,\overset{∙}{q},\ddot{q}$) were computed using an extended Kalman filter (Fohanno et al., 2014) following the segmental coordinate systems defined in the original generic three-dimensional lower limb musculoskeletal model [*Lower Extremity Model*, OpenSim (Delp et al., 1990)]. GRF were smoothed by a 4th order low-pass Butterworth filter with a cut-off frequency of 15 Hz. Raw EMG signals were band pass filtered (4th order) between 30 and 300 Hz, rectified, and EMG envelops were obtained by a 4th order low-pass Butterworth filter with a cut-off frequency of 25 Hz. EMG envelops were then normalized to their respective maximal voluntary activation (Gaudet et al., 2018).

The participant was asked to mimic an equinus gait by producing voluntarily controlled co-contractions of the muscles crossing the ankle joint to restrain ankle dorsiflexion. Eight trials were recorded and the related right steps were analyzed in this study.

### Analysis

In order to evaluate the capacity of the model to reproduce the measured gait pattern and muscle excitations under the mechanical properties and constraints imposed to the model, a set of goodness-of-fit parameters were employed. Root mean square error (RMSE) and coefficient of determination (R^2^) were computed to assess the differences in intensity and shape, respectively, between measured and estimated excitations, joint angles and GRF.

Only estimated muscle excitations corresponding to the measured EMG envelops (Table 2) are presented in this analysis. The coefficient of determination (CC) (Giroux et al., 2013) was computed for the muscles for which EMG data was recorded. This method uses active/inactive state concordance between the estimated muscle excitations and normalized EMG envelopes to compute a coefficient of concordance defined as the percentage of concordance elements.

## Results

**Figure 2:**
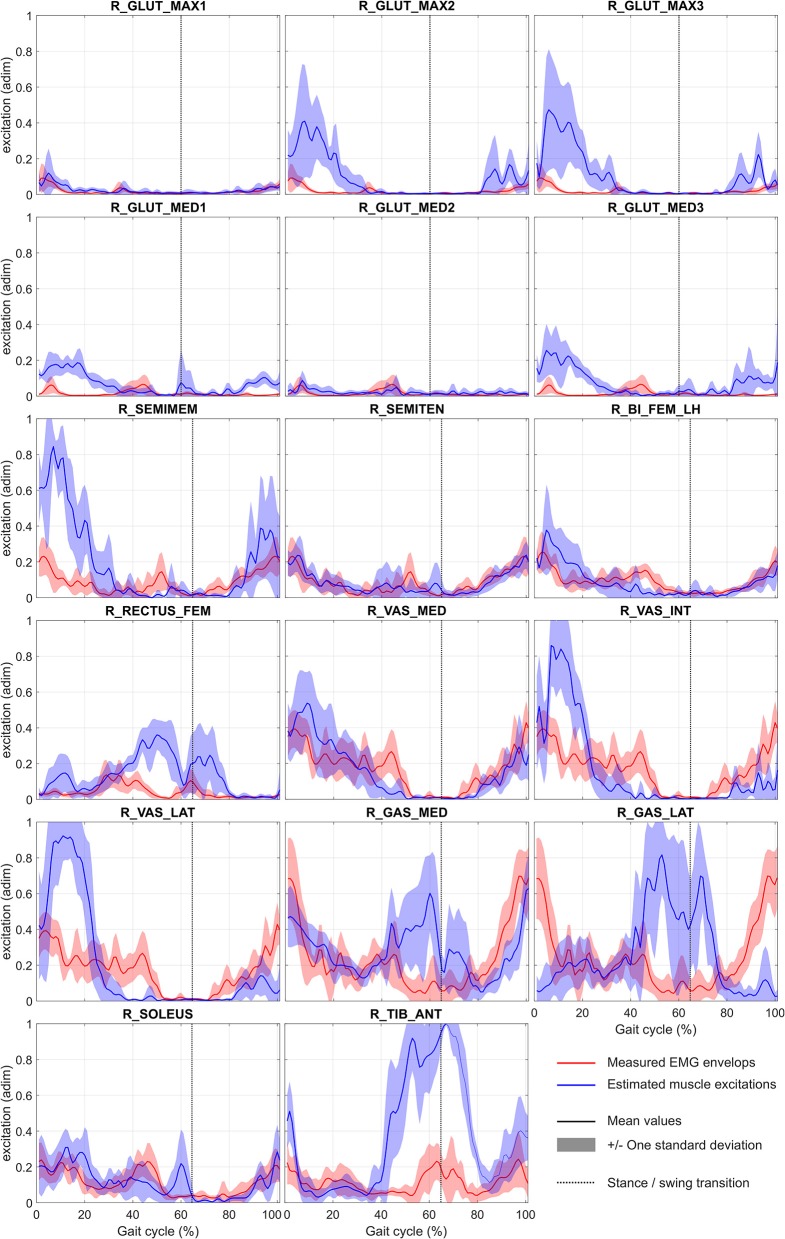
Mean and standard deviation of normalized measured (EMG envelops) and estimated muscle excitations during gait cycle (EMG envelops have been adimensioned (adim) by maximal voluntary contraction). Abbreviations of muscle names are given in Table 2. For illustration purpose, EMG envelops of gluteus maximus, gluteus medius, semitendinosus, and vastus medialis are reported on plots R_GLUT_MAX1/R_GLUT_MAX2/R_GLUT_MAX3, R_GLUT_MED1/R_GLUT_MED2/R_GLUT_MED3, R_SEMIMEM/R_SEMITEN, and R_VAS_MED/R_VAS_INT/R_VAS_LAT, respectively.

**Figure 3:**
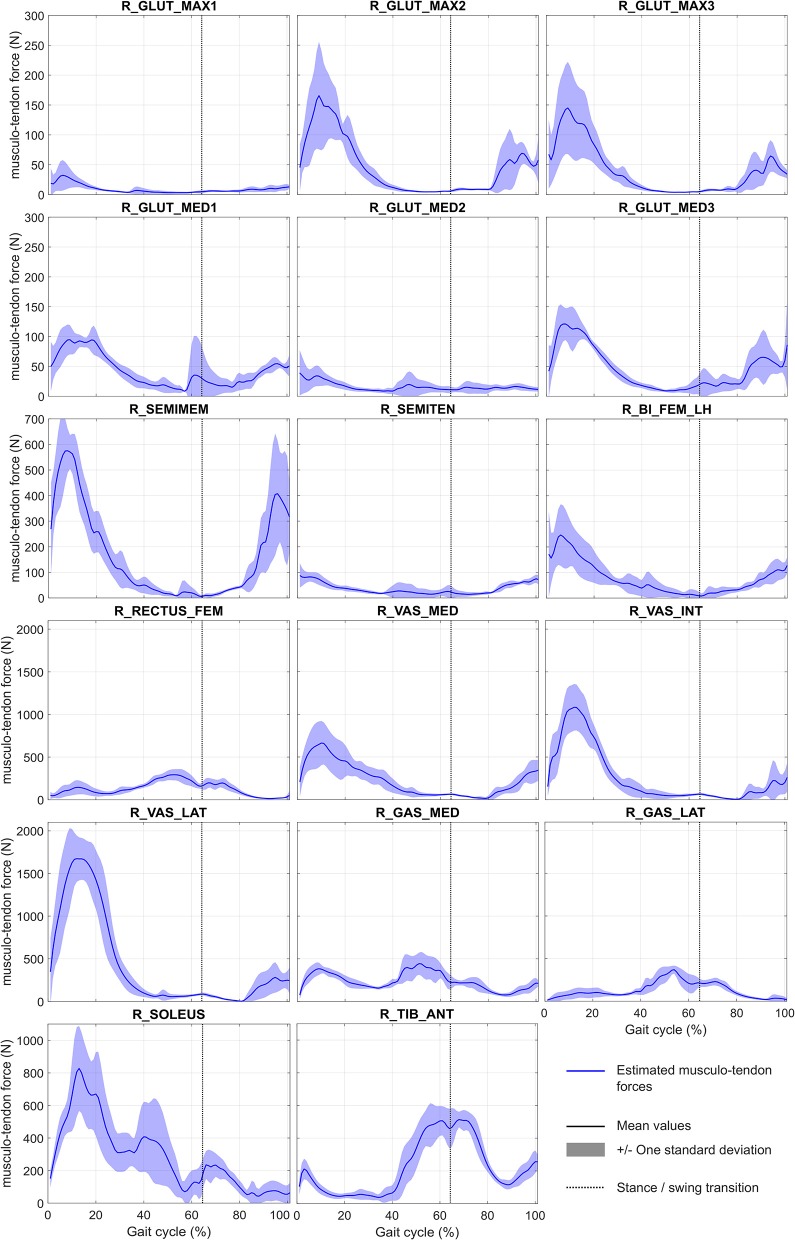
Mean and standard deviation of estimated musculo-tendon forces during gait cycle. Abbreviations of muscle names are given in Table 2.

**Figure 4:**
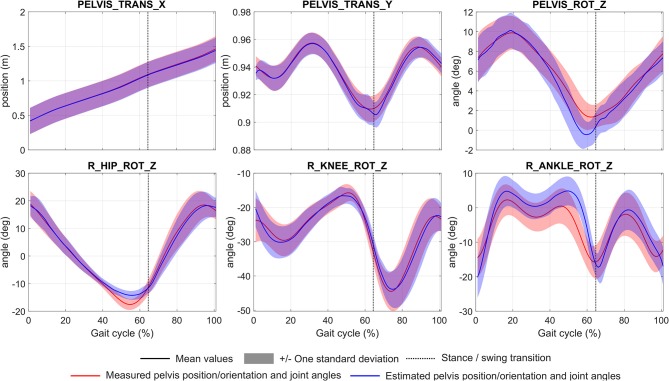
Mean and standard deviation of measured and estimated pelvic position/orientation and joint angles during gait cycle. Abbreviations of each degree of freedom are given in Table 3.

**Figure 5:**
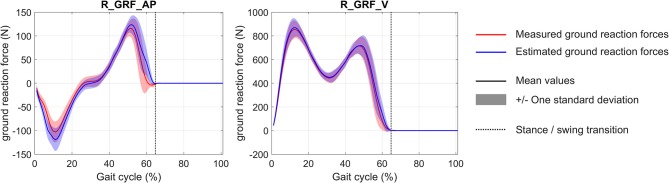
Mean and standard deviation of measured and estimated vertical (R_GRF_V) and anterior/posterior (R_GRF_AP) ground reaction forces during gait cycle.

The convergence time of the eight optimizations using MUSCOD-II was 3.88 ± 1.04 h on an Intel® Core™ i5-3570 CPU @3.4 GHz. Estimated and measured muscle excitations, musculo-tendon forces, joint angles, and GRF are reported in Figures 2–5, respectively. Goodness-of-fit parameters (RMSE, R^2^ and CC) are reported in Table 4.

**Table 4:** Root mean square error (RMSE), coefficient of determination (*R2*) computed to assess the differences in intensity and shape, respectively, between measured and estimated excitations (adimensioned), pelvis position/orientation, joint angles, vertical ground reaction force (R_GRF_V), and anterior/posterior ground reaction force (R_GRF_AP).

	**Cycle 1**		**Cycle 2**		**Cycle 3**		**Cycle 4**		**Cycle 5**		**Cycle 6**		**Cycle 7**		**Cycle 8**	
	**CC (%)**	**80.95**	**CC (%)**	**73.02**	**CC (%)**	**76.19**	**CC (%)**	**85.71**	**CC (%)**	**68.25**	**CC (%)**	**74.60**	**CC (%)**	**76.19**	**CC (%)**	**79.37**
	**RMSE**	**R^**2**^**	**RMSE**	**R^**2**^**	**RMSE**	**R^**2**^**	**RMSE**	**R^**2**^**	**RMSE**	**R^**2**^**	**RMSE**	**R^**2**^**	**RMSE**	**R^**2**^**	**RMSE**	**R^**2**^**
R_GLUT_MAX1	0.02	0.07	0.03	0.37	0.02	−0.11	0.02	0.64	0.05	0.55	0.03	0.13	0.01	0.50	0.03	0.44
R_GLUT_MED2	0.03	0.12	0.04	0.02	0.03	−0.09	0.03	−0.17	0.05	**−2.93**	0.02	**−0.76**	0.01	**−1.13**	0.03	0.21
R_SEMITEN	0.07	0.23	0.14	−0.22	0.06	0.59	0.05	0.24	0.06	0.57	0.07	0.24	0.05	0.64	0.09	0.08
R_BI_FEM_LH	0.06	0.02	0.08	0.10	0.11	0.38	0.07	0.22	0.17	0.17	0.14	0.10	0.06	0.47	0.08	0.27
R_RECTUS_FEM	0.13	0.02	0.20	−0.01	0.18	−0.06	0.15	−0.01	0.23	0.00	0.13	0.03	0.12	−0.09	0.15	0.09
R_VAS_MED	0.10	0.36	0.12	**0.40**	0.13	0.51	0.13	0.18	0.28	0.26	0.14	0.17	0.11	0.50	0.15	0.45
R_GAS_MED	0.16	**−0.70**	0.28	−0.28	0.28	**−0.73**	0.27	**−0.72**	0.34	−0.52	0.27	−0.52	0.22	−0.55	0.26	**−1.07**
R_SOLEUS	0.12	−0.17	0.13	−0.17	0.13	0.14	0.07	0.51	0.14	0.25	0.12	0.30	0.07	0.16	0.08	0.48
R_TIB_ANT	**0.25**	0.24	**0.46**	−0.02	**0.38**	0.05	**0.46**	0.10	**0.51**	−0.03	**0.48**	−0.04	**0.44**	0.07	**0.45**	−0.01
PELVIS_TRANS_X (mm)	8.67	1.00	7.72	1.00	7.65	1.00	10.22	1.00	8.57	1.00	8.84	1.00	8.58	1.00	8.36	1.00
PELVIS_TRANS_Y (mm)	1.75	0.98	2.17	0.98	2.47	0.98	2.14	0.99	3.18	0.96	2.11	0.98	1.75	0.99	2.57	0.97
PELVIS_ROT_Z (°)	0.60	0.98	1.35	0.84	0.66	0.94	1.19	0.88	0.89	0.94	0.75	0.93	0.94	0.92	0.89	0.95
R_HIP_ROT_Z (°)	1.16	0.99	2.09	0.96	1.02	0.99	2.12	0.97	1.57	0.99	1.28	0.99	1.48	0.98	1.15	0.99
R_KNEE_ROT_Z (°)	0.65	0.99	1.13	0.98	1.09	0.97	1.50	0.97	1.53	0.98	1.45	0.98	1.43	0.97	1.84	0.97
R_ANKLE_ROT_Z (°)	**2.54**	**0.86**	**3.62**	**0.79**	**4.43**	**0.71**	**3.46**	**0.76**	**5.48**	**0.66**	**4.15**	**0.73**	**3.33**	**0.79**	**4.75**	**0.74**
R_GRF_AP (N)	11.92	**0.97**	10.95	**0.98**	11.02	**0.95**	13.16	**0.94**	13.43	**0.94**	13.18	**0.93**	12.23	**0.92**	14.03	**0.94**
R_GRF_V (N)	**27.73**	0.99	**21.85**	1.00	**19.56**	1.00	**20.62**	1.00	**21.57**	1.00	**26.18**	0.99	**19.25**	1.00	**19.79**	1.00

*The analysis of muscle excitations is completed by the coefficient of concordance (CC). The results are reported for each gait cycle. Abbreviations of each muscle names and degree of freedom are given in Tables 1, 2, respectively. Bold values correspond to higher RMSE values and lower R2 values*.

Considering the tracked muscle excitations (i.e., muscles for which EMG records are available, see Table 2), the temporal muscle activity of the model was in good overall agreement with the experimental measurements, with an average CC of 76.8 ± 5.3%. RMSE values were generally low with an average value of 0.2 ± 0.1 (values were adimensioned between 0 and 1). However, RMSE was found to be higher for the gastrocnemius medialis (0.3 ± 0.1) and the tibialis anterior (0.4 ± 0.1). For all muscles, the correlation remained low with an average R^2^ at 0.02 ± 0.52. Regarding all other model muscles, for which EMG was not tracked, the optimized muscle excitations were higher than those estimated for muscles for which EMG was tracked. This is the case for R_GLUT_MAX2 and R_GLUT_MAX3 compared to R_GLUT_MAX1, R_GLUT_MED1, and R_GLUT_MED3 compared to R_GLUT_MED2, R_SEMIMEM compared to R_SEMITEN, R_VAS_INT, and R_VAS_LAT compared to R_VAS_MED, and for R_GAS_LAT compared to R_GAS_MED. The same results are observed on the estimated musculo-tendon forces. These forces are ranged between 0 and 2,000 N, with the highest peak forces obtained for R_SEMITEN, R_VAST_INT, R_VAST_LAT, and R_SOLEUS.

With regard to the pelvis position/orientation and joint angles, the model estimations were generally in agreement with the experimental measurements. Average RMSE were 5.4 ± 3.3 mm for the pelvic translations, and 1.9 ± 1.3° for pelvic rotations and joint angles. However, RMSE was found to be higher for the ankle joint (4.0 ± 0.9°). Considering all degrees of freedom, the correlation remained very high with an average R^2^ at 0.94 ± 0.09.

For the GRF, the model estimations were found to be in agreement with the experimental measurements (the average RMSE is 17.3 ± 5.5 N). For these forces, the correlation remained high with an average R^2^ at 0.97 ± 0.03.

## Discussion

The main objective of this study was to adapt an EMG marker tracking optimization process to solve a forward dynamics problem on a 3D musculoskeletal model of the lower limb during equinus gait. To the best of our knowledge, the use of a direct multiple shooting algorithm on a musculoskeletal model with the tracking of measured EMG, marker trajectories, and GRF has never been performed to date. As already shown by Bélaise et al. (2018a,b), this approach allows for an accurate reproduction of joint kinematics and ensures temporal fidelity in muscle activity with improved computational time compared to traditional forward dynamic approaches. We have also highlighted that the tracking of GRF could be performed accurately, even without the use of a complex foot/ground contact model.

### Limitations

A primary limitation of this preliminary study is that it was based on a small number trials for a single task, performed by a single participant. As such, limited conclusions can be drawn from this paper.

A second limitation is that only one contact point was defined at the forefoot and it was only constrained to null velocity and acceleration during the whole contact phase. While this approach was in line with an equinus gait and was able to accurately reproduce the tracked GRF, this definition cannot be applied during normal gait trials, during which several contact points should be defined (Fluit et al., 2014a). Elastic contact elements (Peng et al., 2018), artificial muscle-like actuators (Fluit et al., 2014a) or distance and velocity-dependent force models (Jung et al., 2016) should be adapted to the present model to extend its use to normal gait.

The proposed musculoskeletal model also only consisted of the pelvis and the right lower limb. A forward dynamic approach was made possible by replacing the forces and moments produced by the opposite lower limb and the upper part of the body by a set of generalized forces acting on the pelvis. With such an approach, the individual contribution of the opposite lower limb and the upper part of the body to the muscle activity and joint contact forces of the right lower limb cannot be evaluated individually. It would thus be an important next step to complete the missing body segments of the present musculoskeletal model in order to obtain a full body musculoskeletal model. Several full body musculoskeletal models (Rajagopal et al., 2016; Bassani et al., 2017) have been proposed in the literature and could be transferred to the *bioRBD* musculoskeletal modeling package. These segments could be actuated by joint torques instead of muscles.

In addition to this, while we used a 3D musculoskeletal model, DoFs were reduced to only allow a two-dimensional motion in the sagittal plane. It is however, established that walking is a locomotion task that is performed in sagittal, coronal, and transversal planes (Perry and Burnfield, 2010). In particular, patients often develop compensatory movements in the coronal plane when pathological impairments result in reduced foot clearance capacity in the sagittal plane (Chantraine et al., 2016). Despite this simplification, the accuracy of kinematic tracking observed in the present results suggest that there is potential for the EMG marker tracking optimization process to perform 3D gait motion simulations.

Finally, most of the parameters of the Hill-type muscle model were kept with generic property definitions in this study. Only optimal maximal isometric forces were identified during dynamic optimizations, while muscle optimal lengths, tendon slack lengths, and maximal isometric forces are usually identified in similar studies (Sartori et al., 2014; Pizzolato et al., 2015). This may explain the high excitations observed in muscles contained in a group, for which tracking of excitation was applied to other muscles. The introduction of further muscle parameters could be implemented in future studies, and would only be expected to impact the convergence time of the simulations.

### Muscle Activity

The present study support the results of Bélaise et al. (2018a,b), which demonstrated that EMG tracking could be an efficient way to reproduce measured muscle excitations in simulations. While some amplitude differences appeared for certain tracked muscles, the temporal activity was generally reproduced with good fidelity. This outcome is crucial to ensure the ability of the model to produce muscle co-contractions. Similar approaches have already been proposed in the literature–EMG-driven musculoskeletal models have also been used to accurately reproduce muscle excitation patterns observed on EMG records (Shao et al., 2009; Sartori et al., 2012). However, these models are constrained to have as many muscle lines of action as EMG available in the dataset. To overcome this limitation, some authors have proposed hybrid approaches that combine EMG-driven and static optimization methods (Lloyd and Besier, 2003; Moissenet et al., 2014; Sartori et al., 2014). The drawback with this strategy is that by minimizing the difference between the motor joint moments computed by EMG-driven and inverse dynamics, kinematics may not be accurately reproduced. In that sense, the EMG-marker tracking algorithm proposed by Bélaise et al. (2018a,b) is a novelty. As this method tracks joint kinematics based on marker trajectories rather than joint moments as in a forward dynamic approach, the error diffusion is minimized and the simulation outputs reproduce the experimental kinematics more faithfully.

In our trials of emulated equinus gait, co-contractions of the ankle dorsiflexors, and plantarflexors can be observed during early stance to stabilize the joint in this specific posture. It is interesting to observe that, while the gastrocnemius medialis and the soleus (muscles for which EMG records were tracked) were contracted during this phase, the gastrocnemius lateralis (a muscle for which EMG records were not measured) was not in our simulations. Although use of EMG is somewhat limited to available hardware, muscle locations and the signal quality, measurement should be prioritized toward muscles presumed to be active during the task being investigated. In our case, focusing on a greater number of muscles crossing the ankle joint would have brought more relevant information to the model. A similar recommendation has already been proposed by Sartori et al. (2014); these authors suggested to prioritize EMG use on muscles “*that reflect the patient's non-physiological muscular behavior*.”

Although the EMG-GRF-marker tracking algorithm was able to reproduce physiological muscle activity, two points must be considered. First, we observed that when EMG was not tracked, the optimized muscle excitations and musculo-tendon forces were higher than the ones estimated when muscle EMG was tracked. As pointed out in the limitations of the study, this over-estimation is perhaps related to the use of generic muscle model parameters. For example, if the parameters applied to a muscle group would tend to limit its capacity to produce a motor joint moment, a higher muscle excitation would be required to reproduce the experimental measurements. This effect is further exacerbated if the excitation of a muscle in this group is constrained to a low level in accordance with the experimental EMG tracking, as the excitation of the other muscles of the group will have to compensate for this reduced excitation in the other muscle. Second, due to equinus (results in increased plantarflexion), the capacity of the triceps surae to produce a plantarflexion moment is reduced (Delp et al., 1990), in particular during the push-off phase. Thus, hip flexor recruitment may be increased in this gait pattern to pull the leg forward (Romkes and Schweizer, 2015). However, in our model the primary hip flexors, i.e., the iliopsoas muscles, were not included. van der Krogt et al. (2012) showed that an increased activation of the rectus femoris may be developed to compensate for a weakness of the primary hip flexors. In our case, the absence of the iliopsoas muscles (equivalent to a complete reduction of strength in these muscles) may have induced the increased rectus femoris excitations observed during the simulations compared to the experimental measurements. Because the rectus femoris is a bi-articular muscle (i.e., hip flexor, knee extensor), an increased excitation of this muscle used to assist in hip flexion would simultaneously act to reduce knee flexion, which would have require compensation from knee flexors in order to maintain experimental kinematics (van der Krogt et al., 2012). This could explain the non-physiological activity of the triceps surae (ankle plantarflexors) during pre-swing and early swing observed in our simulations, and the increased tibialis anterior activity (ankle dorsiflexor) used to balance ankle flexion due to the increased activity of ankle plantarflexors. All these observations support the need for a more comprehensive, full body musculoskeletal model, as already discussed in section Limitations.

### Kinematics and Ground Reaction Forces

Unlike inverse dynamics-based optimization approaches (i.e., static optimization), where measured kinematics and calculated joint torques are the input constraints to the optimization problem, in a forward dynamics approach it is essential to assess the accuracy of reproduced kinematics. This is generally assessed by tracking experimental kinematics (Erdemir et al., 2007; Chèze et al., 2015). In the present study, marker trajectories were tracked rather than joint kinematics, as proposed by Bélaise et al. (2018a,b). This tracking was able to produce accurate marker trajectories as highlighted in Bélaise et al. (2018a) with a tracking residual of 0.31 ± 0.32 cm during elbow flexion. This approach, used in the present study gave accurate kinematics with a maximum RMSE obtained at the ankle joint < 5°, a threshold recognized as critical for clinical interpretation (McGinley et al., 2009). The main errors appeared at the end of the stance phase in most of the DoFs. This issue may be associated with the non-physiological activity of the triceps surae and the rectus femoris during this phase, as discussed in section Muscle Activity. Our simulations tended to reduce the plantarflexion induced by the emulated equinus gait. By increasing ankle dorsiflexion, the triceps surae moment arm was increased (Delp et al., 1990) and a minimal ankle plantarflexion moment was kept.

Interestingly, GRF were estimated accurately without an advanced foot-ground contact model definition. The use of simple generalized forces applied on the pelvis, designed to compensate for the absence of the opposite lower limb and the upper part of the body in our model, acted as efficient reserve actuators (Modenese et al., 2013) to provide the forces required to track experimental GRF. This approach may thus present a valid means by which to manage external forces and moments in the dynamic equation when the interactions between the upper limb and/or the contralateral lower limb with the ipsilateral lower limb are not known. Otherwise, as already discussed in section Limitations, a full body musculoskeletal model would be recommended.

### Clinical Perspectives

The simulation of equinus gait represents an important clinical issue in the context of toe walking, a common gait deviation observed in many pathologies such as cerebral palsy, myopathy, and neuropathy (Armand et al., 2007). Numerical simulations may present a useful tool in this context of identifying potential biomechanical causes of this deviation, such as: pre-tibial muscle weakness, inadequate ankle dorsiflexors activity, ankle plantarflexors contracture, and/or spasticity, excessive voluntary ankle plantarflexion in compensation for quadriceps weakness, knee flexor contracture caused by overactivity of the hamstring, combined spasticity of the hamstring and ankle plantarflexors, and leg length discrepancy (Armand et al., 2007; Perry and Burnfield, 2010). In more general terms, there is a need for a numerical framework allowing for the introduction of pathology (Santos et al., 2017), treatment, or surgical intervention-related (Fox et al., 2009) perturbations in a model, and the analysis of their impact on the structures of the musculoskeletal system during daily living activities. However, before clinical applications, models have to be evaluated and validated (Hicks et al., 2015). It will thus be necessary that we assess the capacity of our approach to produce physiological musculo-tendon forces and joint contact forces. Validation datasets, such as the ones made available by Bergmann et al. (2016) and Fregly et al. (2012), should thus be tested on our numerical framework in the future.

## Conclusion

In conclusion, we have improved the recent EMG-marker tracking optimization method to a multiphase cyclic movement with GRF. This numerical framework was successfully tested on a dataset of equinus gait for which our approach was able to estimate lower-limb kinematics, GRF and muscle activity with reasonable accuracy.

## Data Availability

The dataset used for this study is available from figshare (https://figshare.com/s/ddef4ef25acc2ea91c04, doi: 10.6084/m9.figshare.7869293). They are all stored in c3d file format (https://www.c3d.org).

## Ethics Statement

The participant gave written informed consent prior to his inclusion and the protocol was conformed to the Declaration of Helsinki and approved by the National Research Ethics Committee of Luxembourg (201805/01).

## Author Contributions

FM conceived of the presented idea and adapted the theory to the present project. MB, BM, and CB developed the theory. FM and EP performed the computations. MB and BM verified the methods. All authors discussed the results and contributed to the final manuscript.

### Conflict of Interest Statement

The authors declare that the research was conducted in the absence of any commercial or financial relationships that could be construed as a potential conflict of interest.
